# Atomic scale insights on the growth of BiFeO_3_ nanoparticles

**DOI:** 10.1038/s41598-022-08687-y

**Published:** 2022-03-19

**Authors:** N. S. Parvathy, R. Govindaraj

**Affiliations:** 1 Materials Science Group, Indira Gandhi Centre for Atomic Research, HBNI, Kalpakkam, Tamil Nadu 603102 India; 2 Homi Bhabha National Institute, Training School Complex, Anushaktinagar, Mumbai, 400094 India

**Keywords:** Materials science, Physics

## Abstract

This study provides new insights on the formation of the nanocrystallites of phase pure BiFeO_3_ prepared using sol–gel method with tartaric acid as the fuel as comprehended based on the local structure and magnetic hyperfine fields at Fe sites using Mossbauer spectroscopy. Important steps involved in the growth of the nanocrystallites of BiFeO_3_ in the sol–gel reaction are elucidated in a detailed manner in this study for the first time. Three important stages with the second stage marked by the formation of as high as 75% of nanocrystallites of BiFeO_3_ occurring over a narrow calcination temperature interval 700–723 K have been deduced in this study. Variation of hyperfine parameters with calcination temperature of the dried precursor gel leading to an increase in the mean size of crystallites of BiFeO_3_ has been deduced. The nanoparticles of BiFeO_3_ are deduced to exhibit weak ferromagnetic property in addition to being strongly ferroelectric based on the magnetization and P-E loop studies. Consequently an appreciable magneto electric coupling effect in terms of significant changes in P-E loop variation with the application of external magnetic field is elucidated in this study, which is comprehended based on the defects associated with BiFeO_3_ nanoparticles.

## Introduction

BiFeO_3_ continues to be one of the most important functional materials in terms of ferroelectric and magnetic properties at room temperature^1–6^. Although there have been a large number of important work carried out on various aspects of multifunctional properties of BiFeO_3_^7–12^, two core issues such as the understanding of the nucleation and growth of BiFeO_3_ and the ways to invoke strong magnetoelectric coupling effects are still remaining important and are being extensively addressed by researchers^10,13–19^. Preparation of BiFeO_3_ by means of solid state reaction method is mostly reported to lead to the presence of other thermodynamically stable impurity phases such as Bi_25_FeO_40_, Bi_2_Fe_4_O_9_ which is understood to be mainly due to the volatility of Bi^20,21^. Bulk BiFeO_3_ is antiferromagnetically ordered and has been reported to display little or very less value of magneo electric coupling effects^22–27^, while thin film of BiFeO_3_ coated on some suitable substrates are reported to exhibit weak ferromagnetic properties^23,28–32^. A large number of work carried out on the nanoparticles of BiFeO_3_ are shown to exhibit weak ferromagnetic properties^33–39^, but with these particles exhibiting only partial or totally lacking of ferroelectric properties. Though quite a few of the research work carried out in nano BiFeO_3_ illustrate the occurrence of the ferroelectric in addition to weak ferromagnetic properties^40–42^, a detailed comprehension of this is yet to emerge which would be quite useful in fine tuning the multiferroic properties of nano BiFeO_3_ leading to significantly enhanced device applications .

Nanoparticles of BiFeO_3_, as compared to that of the bulk analogue, are prepared at lower synthesis temperature around 700 K with the phase purity and the mean size of the particles being crucially dependent upon the fuel used in the chemical method and heat treatments. But even in the case of certain chemical methods of preparation of nanoparticles of BiFeO_3_, presence of Bi_25_FeO_40_, Bi_2_Fe_4_O_9_ has been reported^43–46^. This implies that a further understanding of the nucleation of nano crystallites and growth of BiFeO_3_ might be needed to obtain phase pure BiFeO_3_ nanoparticles of desired size and to fine tune magnetic and the multiferroic properties. In any chemical method of preparation the carboxyl group are required to obtain a homogenous polyester complex of Bi^3+^ and Fe^3+^ ions whereas hydroxyl groups are necessary for further polysterification of carboxyl groups. Thus, the choice of chelating compound influences the structure of the metal complex^44,47,48^. and in addition plays an important role in the phase purity, controlling shape, size distribution and crystalline nature of the products^43–45^. Phase pure BiFeO_3_ nanoparticles were reported to have been prepared using sol–gel method with tartaric acid as the fuel^33,43,44,46,49^, subsequent to calcination close to 700 K, though the dried precursor as obtained due to the reaction was reported to be of amorphous phase as deduced using XRD.

The present work for the first time is aimed at providing a detailed atomic scale understanding of the nucleation and growth of BiFeO_3_ by means of probing the local structure and magnetic fields at Fe sites using Mössbauer spectroscopy^50,51^. This study also elucidates the important role being played by bismuth iron tartarate based polydentates towards obtaining phase pure BiFeO_3_ following calcination at elevated temperatures. Further this work provides the atomic scale understanding of the weak ferromagnetic and significant magneto electric coupling effects in the nanoparticles of BiFeO_3_ as being reported first in this study.

## Experimental methods

### Sample preparation

Bi(NO_3_)_3_·5H_2_O and Fe(NO_3_)_3_·9H_2_O of stoichiometric amounts were dissolved in 2 N HNO_3_ solution by rigorous stirring Tartaric acid (TA) is used as chelating agent in this synthesis procedure. TA is added to the solution keeping the molar ratio of TA to the total metal nitrates as 1:1. In the present case the gel solution was heat treated at 363 K for 4 h mainly towards dehydration. Subsequently the temperature was raised to 423 K and was held for 3 h to enhance the rate of evaporation leading to the formation of dried powder. The resultant powder has been subjected to calcination treatments at different selected temperatures from 573 to 873 K, with each calcinating step time extended for 2 h. Dried gel is found to be magnetically attractive which is observed to get decreased with increasing calcination temperature. The bulk structural, magnetic and ferroelectric properties have been studied in the dried gel subjected to different annealing treatments leading to the formation of the nanoparticles of different phases and with varying size.

### Characterization

#### Structural characterization

The crystal structure and phase purity of the as dried powder were determined by X-Ray diffraction (XRD) using Cu Kα source with diffracted angular range of 2θ from 20° to 90° with a step size of 0.015. Following this the dried powder was subjected to calcination selectively at different temperatures for 2 h and the XRD analysis were carried at room temperature. Reitveld refinement of the XRD pattern has been done using GSAS software.

#### Microstructural characterization

Microstructural studies were done on these samples using Scanning electron microscopy (SEM) (Zeiss sigma 300) mainly to analyze particle size and composition of the samples.

#### Bulk magnetic properties

The magnetic characterization of the samples have been carried out based on the magnetization studies using Vibrating Sample Magnetometer (VSM) from M/s. Cryogenic Inc. Magnetization as a function of temperature was acquired in two different modes viz*.,* zero field cooled (ZFC) and field cooled (FC) protocols with applied field of 500Oe. Variation of the magnetization of the experimental samples with the applied magnetic field has been obtained at 4 K and at 300 K using the VSM.

#### Ferroelectric studies

The ferroelectric properties of the samples have been studied using P-E loop tracer (Marine India). Magneto electric coupling effects are studied directly by means of carrying out P-E loop studies under the application of external magnetic field.

#### Local structural and magnetic properties

^57^Fe based Mössbauer studies have been carried out using ^57^Co dispersed in Rh matrix . Mössbauer spectrometer (Wissel make) was used in constant acceleration mode and in transmission geometry. The values of isomer shift given in this study are deduced with respect to α-Fe absorber at 300 K. Each spectrum is fitted with Lorentzian line shapes of line width Γ_i_ using a nonlinear least—squares program to obtain hyperfine parameters such as isomershift (δ_i_), quadrupole splitting (Δ_i_) and magnetic hyperfine fields (B_hf_) as experienced by different fractions (f_i_) of Fe absorber atoms.

## Results and discussion

### Structural and morphological studies

**Figure 1:**
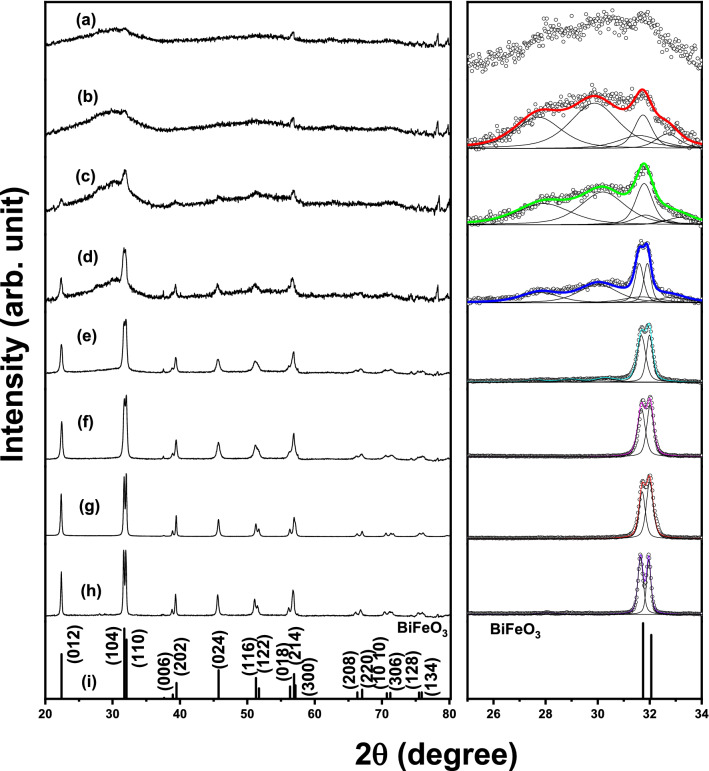
XRD patterns as obtained in (a) dried gel and following the calcinations of the gel in air for 2 h at the following temperatures viz., (b) 573 K, (c) 673 K, (d) 693 K, (e) 708 K, (f) 723 K, (g) 773 K and (h) 873 K and (i) stick pattern of BiFeO_3_.

XRD pattern (Cf. Fig. 1) corresponding to the dried precursor prepared through sol- gel method with tartaric acid as the fuel, resembles that of any typical amorphous system with a broad asymmetric peak over the region of 2θ extending from 20° to 40° with the peak coinciding with the most intense peak position corresponding to BiFeO_3_. However the diffraction peaks observed for the values of 2θ beyond 45° are found to be matching with that of BiFeO_3_. Also an intense peak corresponding to Bi_25_FeO_40_ is observed at 2θ close to 80°. While the pattern obtained subsequent to the annealing treatment at 573 K remains almost same as that of the precursor, peaks corresponding to that of the BiFeO_3_ phase are seen to emerge well above amorphous like background in the pattern corresponding to the sample annealed at 673 K. A sharp decrease in the background is observed subsequent to annealing at 693 K concomitant with the appearance of all the peaks of BiFeO_3_ phase marking the onset temperature of formation of nanocrystallites of BiFeO_3_ as deduced by XRD. Three important observations are made based on the analysis of XRD patterns viz., (1) the appearance of sharp peaks corresponding dominantly to BiFeO_3_ and that of Bi_25_FeO_40_ phases at high values of 2θ beyond 50 degrees (2) the broad peak which appears between 20° and 40° in the patterns (a–d) is quite asymmetric extending more towards lower 2θ values with the peak position matching close to that of BiFeO_3_ and (3) the sharp peaks corresponding to impurity phases disappear following annealing treatments beyond 700 K. These points imply the presence of nanoparticles of other impurity phases causing the appearance of the broad amorphous like feature in the XRD patterns corresponding to samples (a–d). On the basis of these important observations, the broad peak as obtained in the samples (a–d) is de convoluted in to several pseudo-voigt functions to identify the impurity phases that could be present as nanoparticles in the sample. This is illustrated in the XRD patterns as shown on the right side panel of Fig. 1. Panel shown on the right hand side of Fig. 1 contains the XRD patterns corresponding to 2θ range between 20 and 40°, de convoluted in to several pseudo-voigt functions. These results imply the presence of nanoparticles of the impurity phases such as Bi_25_FeO_40_ and α-Fe_2_O_3_ in addition to the dominantly present BiFeO_3_ (Fig. S1). Pattern obtained in the precursor annealed at different temperatures above 700 K imply the occurrence of only BiFeO_3_ phase with the peaks indexed for the rhombohedral structure.

**Figure 2:**
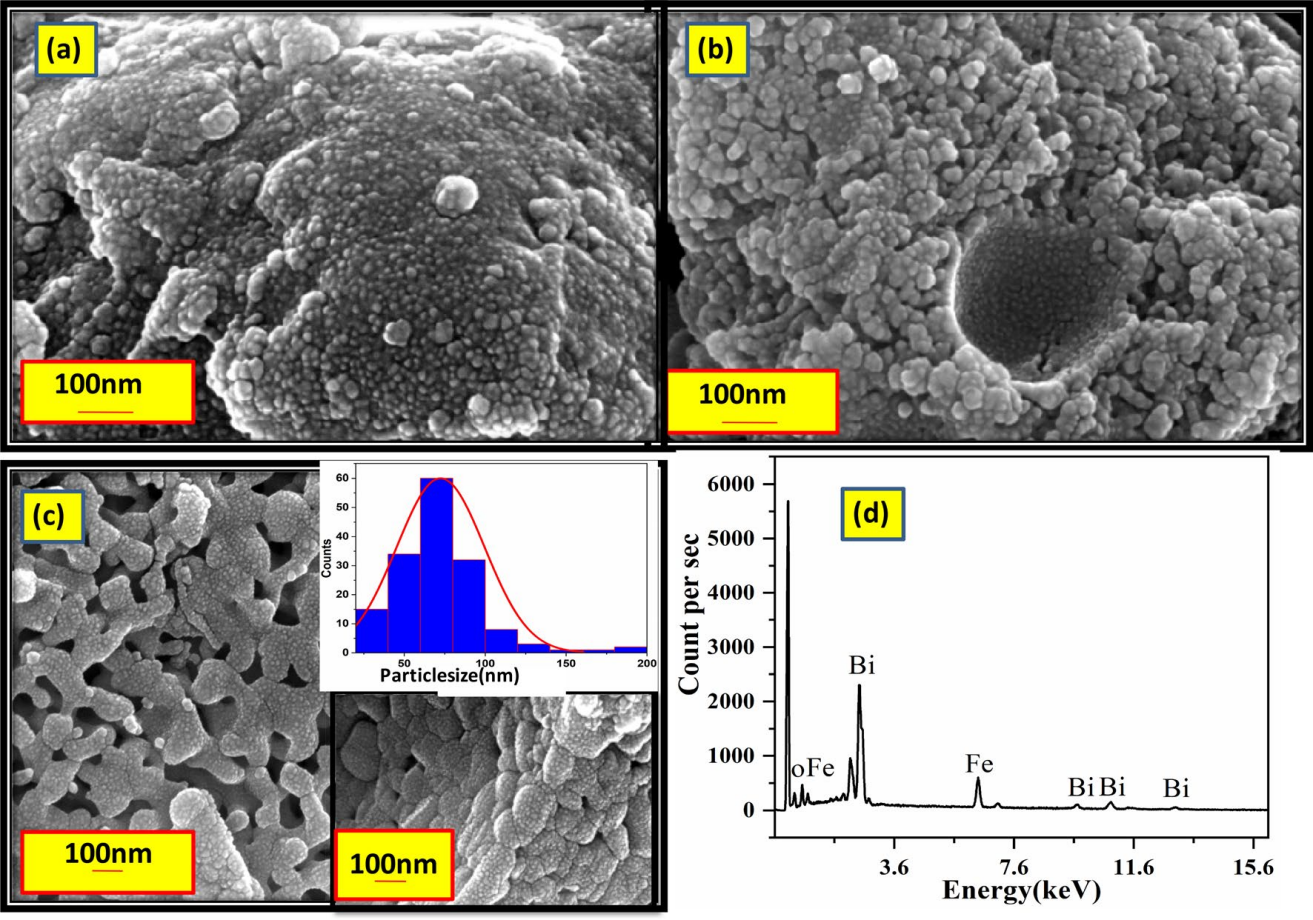
SEM images of the precursor annealed at (**a**) 573 K 2 h, (**b**) 673 K 2 h, (**c**) 773 K 2 h. Shown in inset of (**c**) are the SEM micrographs taken with low and high magnification along with the result of the particle size distribution analysis showing that the mean particle size is around 70 nm. While the EDAX spectrum (Cf. **d**) obtained in the precursor annealed at 773 K shows the peaks corresponding to Bi and Fe showing that the atomic ratio of Bi and Fe as 1:1.

FE SEM images of the precursor subsequent to calcinations at 573 K, 673 K and 773 K are shown in the Fig. 2. Annealing of the precursor at 573 K is observed to result in very fine nanoparticles dominatly of BiFeO3 as elucidated based on EDAX results (Cf. Fig. S2). The formation of lumps and beads shaped structures comprising of aggregation of nanoparticles are seen subsequent to calcination at 673 K. Interestingly the deep pit like structures as seen in SEM image (Cf. Fig. 2b) are understood to have got developed following the emission of gases due to the dissociation of the polydentate based structures that have got formed in the precursor gel due to the sol–gel reaction. SEM micrograph corresponding to the precursor annealed at 773 K shows the presence of the agglomeration of the nanoparticles of BiFeO_3_. Particle size distribution analysis based on SEM results as obtained in the precursor subjected to annealing at 773 K (Cf. inset of Fig. 2c) shows that the mean particle size is close to 70 nm. From the EDX spectrum corresponding to this sample as shown in Fig. 2d it is deduced that the composition ratio of Bi to Fe is 1:1, implying the stoichiometric nature of BiFeO_3_ phase.

### Bulk magnetic and ferroelectric properties

**Figure 3:**
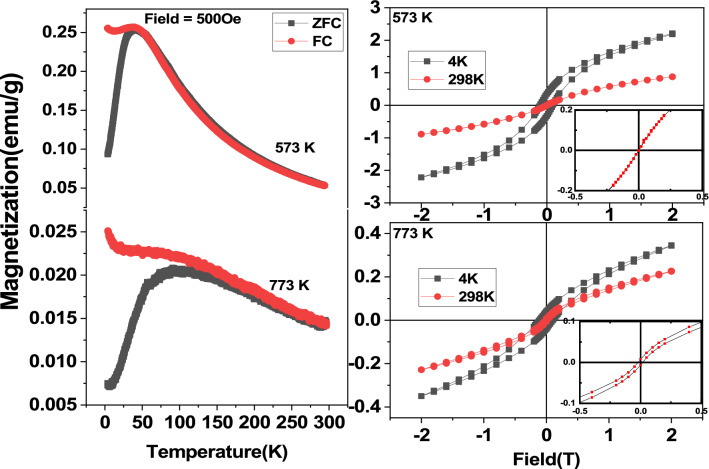
Temperature and magnetic field dependence of magnetization are shown corresponding to the precursor calcined at 573 K and 773 K. Variation of magnetization in terms of ZFC and FC with temperature corresponding to the precursor calcined at 573 and 773 K are shown. Variation of magnetization with the applied magnetic fields is shown in the right panel corresponding to the precursor of the sol–gel reaction subjected to calcination at 573 K and 773 K.

Shown in Fig. 3 are the magnetization results in terms of zero field cooled and field cooled magnetization curves for an applied magnetic field of 500 Oe as obtained in the precursor subjected to calcinations at 573 and 773 K respectively. FC-ZFC magnetization curves as obtained following calcination at 573 K (Cf Fig. 3) are observed to be coinciding and thus exhibiting similar change in terms of increase of magnetization which reach maximum value around 50 K. Below which it is observed that the value of ZFC decreases sharply. On the hand the FC curve is observed to slightly decrease and exhibit an increase below 10 K. ZFC curve shows a behaviour typical of a superparamagnetic (SPM) nature of the particle with the blocking temperature of the system lies around 50 K. M-H loop obtained at 298 K subsequent to calcination at 573 K shows that the loop is non-linear with zero area where as the loop obtained at 4 K shows the superparamagnetic nature of the sample. Non-saturation behaviour of the loop implies the presence of competing anti ferromagnetic interactions^33,52,53^. ZFC–FC curves corresponding to the sample annealed at 773 K coincide only beyond the value of the temperature at which ZFC attains the maximum. This implies that there is a distribution in the blocking temperature understood to be arising due to the distribution in the mean size of the BiFeO_3_ nanoparticles. M-H loop corresponding to the precursor annealed at 773 K shows the weak ferromagnetic behaviour of this sample with non-saturating loop implying the presence of competing ferromagnetic interactions in the nanoparticles of BiFeO_3_ which are largely AFM ordered due to core while the uncompensated spins at the shells result in weak ferromagnetic ordering.

**Figure 4:**
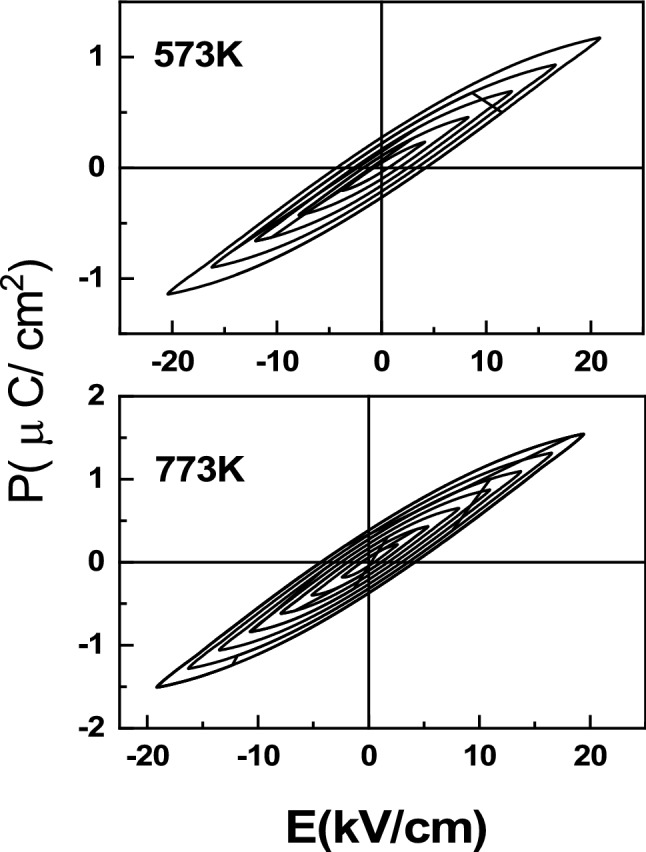
P-E loop as obtained in the precursor gel subsequent to calcination at different temperatures viz*.*, 573 and 773 K respectively.

All the P-E loop measurements have been carried out at room temperature with P-E loop tracer operated at constant frequency of 50 Hz with R and C parameters were set at 100kΩ and 100μF respectively. Due to the difference in the value of the breakdown field of each of these samples, different drive fields were applied (Fig. 4). Saturated P-E loop as obtained in these samples establish the ferroelectric behaviour of the BiFeO_3_ nanoparticles. The ferroelectric parameters such as spontaneous polarization, coercivity, and remnant polarization are measured from the P–E hysteresis loop.

Results of the bulk structural, magnetic and ferroelectric properties are summarized as follows. XRD results mainly imply the presence of nanoparticles of BiFeO_3_ along with nanoparticles of Bi rich and Fe rich phases of bismuth iron oxide in the precursor derived through sol–gel nroute. Very fine nanoparticles of α-Fe_2_O_3_/BiFeO_3_ are established to exhibit weak ferromagnetic ordering due to non-cancellation of spins at the surface and due to interaction between other particles though the core would antiferromagnetically ordered. Non-saturation of M-H loop as deduced in these particles is comprehended due to competing magnetic interactions between core and shell which in turn are significantly influenced by the magnetic interactions between other particles^54^. Saturated P-E loop implies the strong ferroelectric nature of these nanoparticles of the nanoparticles of BiFeO_3_. Having discussed the results of bulk magnetic and ferroelectric properties, in the following the nucleation and growth of the nanoparticles of BiFeO_3_ is elucidated by means of studying the local structure and magnetic fields at Fe sites using Mössbauer spectroscopy subsequent to calcination of precursor at different temperatures.

### Atomic scale understanding of nucleation and growth of BiFeO_3_ using Mӧssbauer spectroscopy

Based on the Mössbauer results reported in the case of the bulk BiFeO_3_^55,56^ it is seen that the Fe atoms associated with FeO_6_ octahedra oriented along <111> are subjected to varying magnitude of rhmbohedral distortion leading to variation in the Fe–O–Fe bond angle. The varying distortion and the Fe–O–Fe angle at these two octahedra thus results in the change in the values of super exchange interaction at the sites of Fe atoms of FeO_6_ located along <111> , thus leading to two distinct Fe sites with equal distribution of the fractions of Fe atoms experiencing different values of quadrupole splitting and magnetic hyperfine fields^55,56^. The presence of nano particles of BiFeO_3_ along with that of α-Fe_2_O_3_ and Bi_25_FeO_40_ could be deduced using XRD (Fig. S1) and SEM analysis. Hence in the present study the fractions of Fe atoms if any were associated with superparamagnetic (SPM) nanoparticles corresponding to antiferromagnetic α-Fe_2_O_3_/BiFeO_3_ would be subjected to pure quadrupole interactions resulting in doublet in the Mössbauer spectrum (MS) as comprehended due to spin fluctuations^48,57–61^. Also the Fe atoms associated with the nanoparticles of the paramagnetic Bi_25_FeO_40_ would result in doublet with the characteristic values of isomer shifts and quadrupole splitting. On the other hand Fe atoms associated with alpha-Fe_2_O_3_/BiFeO_3_ particles of size larger than that of superparamagnetic (SPM) limit would experience weak magnetic hyperfine interaction resulting in the appearance of six line patterns^62–64^ with the values of hyperfine parameters different from that of the bulk which are antiferromagnetically ordered.

**Figure 5:**
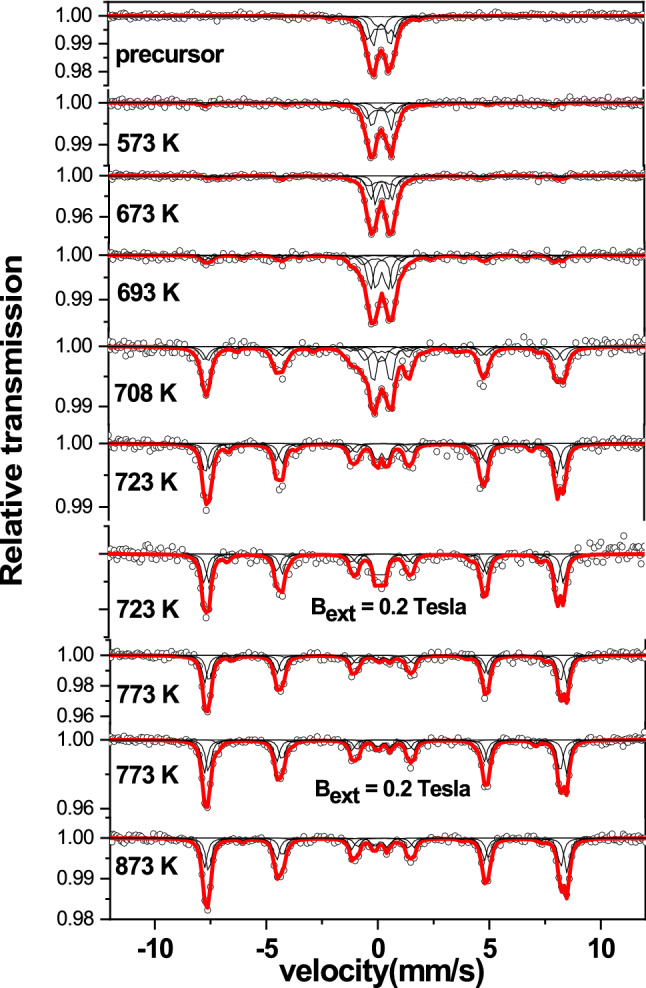
Mössbauer spectra obtained at 300 K in the pristine precursor gel and subsequent to calcination at different temperatures for 2 h as mentioned.

**Figure 6:**
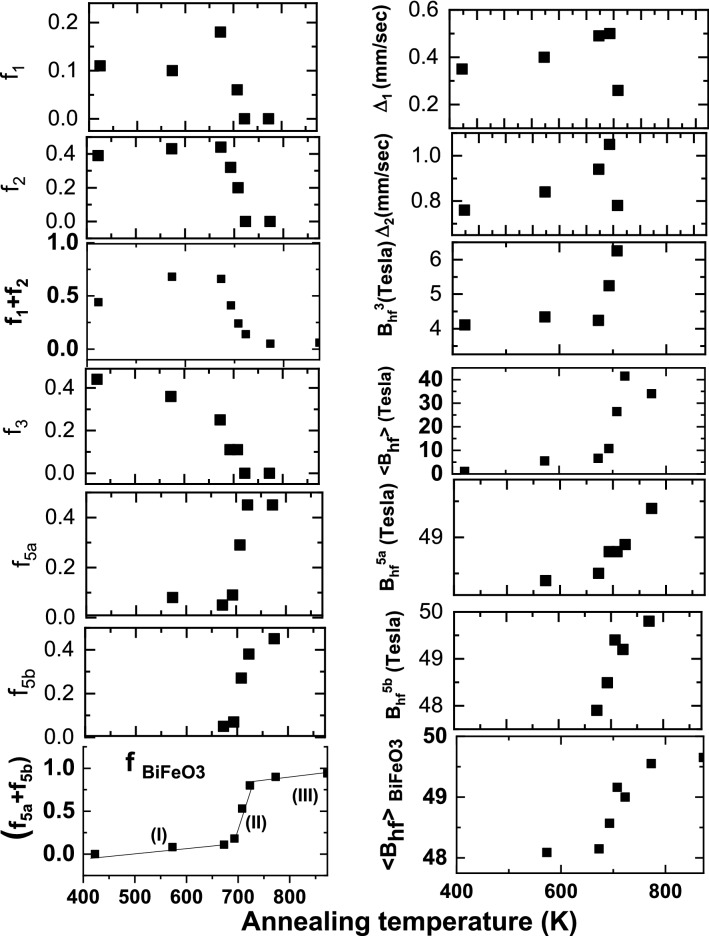
Variation of Mӧssbauer results corresponding mainly to Fe atoms associated with BiFeO_3_ with respect to calcination temperature. Based on the Mossbauer results the stages (I) and (II) are understood to be due to nucleation, growth and the formation of nanocrystalites of BiFeO_3_ respectively. While stage III implies the coarsening of nanocrystallites of BiFeO_3_ in to polycrystalline phase. Mossbauer study brings out that the importance of the stage II leading to the formation of nanocrytallites of BiFeO_3_ in large fraction close to 0.8 along with the elucidation of the evolution of hyperfine parameters such as hyperfine field with calcination temperature in this intervalmarking an increase in crysallite size.

MS spectra obtained at room temperature subsequent to calcination of the precursor at different temperatures are shown in Fig. 5. Results of the analysis of the room temperature Mössbauer studies carried out in the dried precursor gel subsequent to calcination at different temperatures up to 873 K are shown in the Table 1. Important results of the Mössbauer study in terms of the variation of the main hyperfine parameters as the function of calcination temperature are shown in Fig. 6. By means of carefully analyzing these variations the fractions of Fe atoms associated with different sites in the samples are identified as discussed below.

**Table 1:** Results of hyperfine parameters as obtained in the precursor calcinated at different temperatures.

Sample	I	δ_i_ (mm/s)	Δ_i_(mm/s)	B^i^_hf_(Tesla)	f_i_
Precursor	1	0.30 ± 0.03	0.35 ± 0.05	0	0.11
(a)	2	0.36 ± 0.01	0.76 ± 0.01	0	0.39
	3	0.30 ± 0.03	0.06 ± 0.05	4.11 ± 0.07	0.44
	4	0.03 ± 0.08	− 0.21 ± 0.15	12.27 ± 0.45	0.06
573 K 2 h	1	0.33 ± 0.04	0.40 ± 0.16	0	0.10
(b)	2	0.37 ± 0.04	0.84 ± 0.16	0	0.43
	3	0.28 ± 0.02	0.14 ± 0.03	4.34 ± 0.10	0.36
	4	0.32 ± 0.18	0.20 ± 0.33	11.44 ± 1.00	0.03
	5	0.42 ± 0.05	− 0.32 ± 0.11	48.40 ± 0.36	0.08
673 K 2 h	1	0.34 ± 0.01	0.49 ± 0.04	0	0.16
(c)	2	0.32 ± 0.01	0.94 ± 0.07	0	0.42
	3	0.32 ± 0.08	0.017 ± 0.12	4.24 ± 0.20	0.24
	4	0.32 ± 0.18	0.20 ± 0.33	11.44 ± 1.00	0.04
	5a	0.46 ± 0.04	0.53 ± 0.009	47.92 ± 0.31	0.05
	5b	0.40 ± 0.04	− 0.03 ± 0.09	48.60 ± 0.33	0.05
	6	0.22 ± 0.08	0.45 ± 0.15	43.02 ± 0.57	0.04
693 K 2 h	1	0.35 ± 0.01	0.51 ± 0.01	0	0.31
(d)	2	0.35 ± 0.01	1.04 ± 0.01	0	0.32
	3	0.12 ± 0.04	0.52 ± 0.07	5.24 ± 0.23	0.12
	4	0.40 ± 0.08	0.73 ± 0.25	11.11 ± 0.58	0.04
	5a	0.35 ± 0.03	− 0.23 ± 0.07	48.49 ± 0.26	0.09
	5b	0.41 ± 0.04	0.31 ± 0.09	48.81 ± 0.27	0.08
	6	0.40 ± 0.07	0.10 ± 0.14	39.26 ± 0.43	0.04
708 K 2 h	1	0.33 ± 0.04	0.26 ± 0.07	0	0.06
(e)	2	0.35 ± 0.01	0.78 ± 0.02	0	0.20
	3	0.43 ± 0.03	0.18 ± 0.05	6.25 ± 0.19	0.11
	4	0.47 ± 0.11	− 0.01 ± 0.22	19.67 ± 0.70	0.04
	5a	0.36 ± 0.01	− 0.16 ± 0.06	48.83 ± 0.14	0.29
	5b	0.37 ± 0.01	0.32 ± 0.06	49.41 ± 0.14	0.26
	6	0.32 ± 0.09	0.51 ± 0.19	41.75 ± 0.64	0.04
723 K 2 h	1a	0.35 ± 0.01	0.46 ± 0.02	0	0.11
(f)	5a	0.38 ± 0.007	− 0.11 ± 0.01	48.91 ± 0.04	0.45
	5b	0.40 ± 0.009	0.35 ± 0.02	49.16 ± 0.0	0.38
	6	0.36 ± 0.04	− 0.19 ± 0.09	42.06 ± 0.32	0.06
723 K 2 h	1a	0.29 ± 0.02	0.34 ± 0.02	0	0.12
Bext = 0.3 T	5a	0.39 ± 0.007	− 0.17 ± 0.01	48.98 ± 0.04	0.43
(g)	5b	0.41 ± 0.009	0.28 ± 0.02	49.3 ± 0.06	0.38
	6	0.35 ± 0.03	0.2 ± 0.09	43.8 ± 0.32	0.07
773 K 2 h	1a	0.39 ± 0.04	0.49 ± 0.08	0	0.04
(h)	5a	0.38 ± 0.01	− 0.10 ± 0.01	49.40 ± 0.04	0.46
	5b	0.40 ± 0.01	0.33 ± 0.01	49.74 ± 0.03	0.46
	6	0.32 ± 0.04	0.58 ± 0.09	43.80 ± 0.34	0.04
773 K 2 h	1a	0.40 ± 0.02	0.50 ± 0.04	0	0.05
Bext = 0.3 T	5a	0.38 ± 0.004	− 0.12 ± 0.009	49.43 ± 0.02	0.45
(l)	5b	0.40 ± 0.004	0.31 ± 0.01	49.77 ± 0.03	0.46
	6	0.24 ± 0.03	− 0.32 ± 0.06	44.10 ± 0.22	0.04
873 K 2 h	7	0.25 ± 0.01	0.57 ± 0.03	0	0.07
(j)	5a	0.39 ± 0.005	− 0.12 ± 0.01	49.49 ± 0.03	0.41
	5b	0.40 ± 0.004	0.33 ± 0.01	49.82 ± 0.03	0.49
	6	0.39 ± 0.06	0.74 ± 0.12	41.53 ± 0.42	0.03

f_1_ and f_2_ are due to relative fractions of iron atoms associated with nanoparticles of Bi_25_FeO_40_ and superparamagnetic particles (SPM) of BiFeO_3_ and α-Fe_2_O_3_. f_3_ is the fraction of iron atoms associated with nanoparticles of BiFeO_3_ with size larger than the SPM limit and much lesser than that of bulk BiFeO_3_ as deduced based on the hyperfine parameters. f_4_ is the fraction of iron atoms associated with BiFeO_3_ particles of size larger than that of f_3_. f_5a_,f_5b_ are the fractions of iron atoms associated with that of bulk BiFeO_3_ particles, while f_6_ is interpreted to be due to iron atoms associated with shell with core as BiFeO_3_. In the case of precursor annealed at 873 K and beyond the fraction f_7_ is attributed to Fe atoms associated with the shell of Bi_25_FeO_40_ formed at the surface of large sized BiFeO_3_.

Mössbauer spectrum (MS) obtained in the precursor dried at 423 K could be de-convoluted in to two doublets and two six line components with the latter experiencing low hyperfine fields as shown in Fig. 5. The analysis of MS spectrum obtained subsequent to calcination of the dried precursor gel at 573 K shows that there is a slight increase in the value of f_2_ while that of f_1_ remains the same. There is a decrease in f_3_ the fraction of Fe atoms experiencing low hyperfine field close to 4 Tesla by 8%. Interestingly there is an occurrence of a fraction close to 8% of iron atoms exposed to a hyperfine field of 48 Tesla and a quadrupole splitting around − 0.32 mm/s. This value of of 48 Tesla is close to that of the magnetic hyperfine field associated with that of BiFeO_3_^55,56^. This result implies the coalescence of weakly ferromagnetic nanoparticles of BiFeO_3_ leading to the formation of nanocrystallites of BiFeO_3_. This observation is further substantiated with the Mössbauer results obtained in the precursor calcined at 673 K in which the occurrence of two distinct fractions of iron atoms (f_5a_,f_5b_) corresponding to FeO_6_ sites of BiFeO_3_ experiencing different values of quadrupole splitting and magnetic hyperfine fields but close to 48 Tesla could be inferred based on the analysis. Occurrence of the fractions of Fe atoms associated with BiFeO_3_ (f_5a_ + f_5b_) is seen at the cost of the reduction in f_3_ associated with low hyperfine field (4 T) in the temperature interval below 693 K. This implies that the fraction f_3_ is associated with the nanocrystallites of BiFeO_3_ of size just above the superparamagnetic limit. In the temperature interval below 700 K there is a coalescence of the fine nanocrystallites of BiFeO_3_ resulting in the increase of the fraction (f_5a_ + f_5b_) of Fe atoms associated with the fine particles of BiFeO_3_. The coalescence of the fine nanoparticles of BiFeO_3_ is understood to have resulted in the enhanced orientation of crystallites leading to appearance of peaks corresponding to BiFeO_3_ well above the amorphous background as observed in the XRD patterns obtained subsequent to calcination treatment carried out at 673 K and beyond.

It is important to note that the fraction of iron atoms associated with two distinct sites of BiFeO_3_ (f_5a_ + f_5b_) increases from the value of 0.15 to 0.8 in a narrow temperature interval of (700–723 K). The significant decrease in (f_1_ + f_2_) in this narrow temperature interval is therefore closely correlated with a sharp increase in the fraction of iron atoms associated with BiFeO_3_ and hence the formation of nanocrystallites of BiFeO_3_. The value of f_1_ which remains small close to 0.1 in the dried precursor gel is seen to increase for calcination treatments carried out close to 700 K. The value of f_2_ is observed to remain almost constant for calcination treatment upto 700 K and decreases following calcination above this temperature. Hence it is interpreted that the fraction f_1_ is due to Fe atoms associated with Bi richer phase such as Bi_25_FeO_40_. Due to volatility of Bi, there is likely to be a tendency for the Bi richer phase to increase in concentration with increasing calcination temperature. The fraction f_2_ is attributed to Fe atoms associated with superparamagnetic particles of BiFeO_3_ phase dominantly, along with those of α-Fe_2_O_3_. f_4_ is identified as the fraction of Fe atoms associated with slightly larger sized nanoparticles of BiFeO_3_ and get formed at the cost of f_3_ due to magnetic interactions between them. The following are the important results of the Mossbauer studies viz., (i) a slight increase in the fraction of Fe atoms associated with antiferromagnetic BiFeO_3_ to 0.1 below 700 K is ascribed to coalescence of nano BiFeO_3_ particles, (ii) a sharp increase in the fraction of nanocrystallites of BiFeO_3_ from 0.1 to 0.85 in the narrow temperature interval of 700–723 K and (iii) slight increase in the fraction of BiFeO_3_ up to 0.95 in the temperature interval from 730 to 873 K. In the following discussion the comprehension is provided for the occurrence of fractions of Fe atoms associated with the particles representing bismuth iron oxides rich in Bi and Fe respectively along with the fraction associated with BiFeO_3_ particles. Also the understanding of the stage-II marking the sharp increase in the fraction of Fe atoms associated with BiFeO_3_ in the temperature interval 700–723 K is provided.

**Figure 7:**
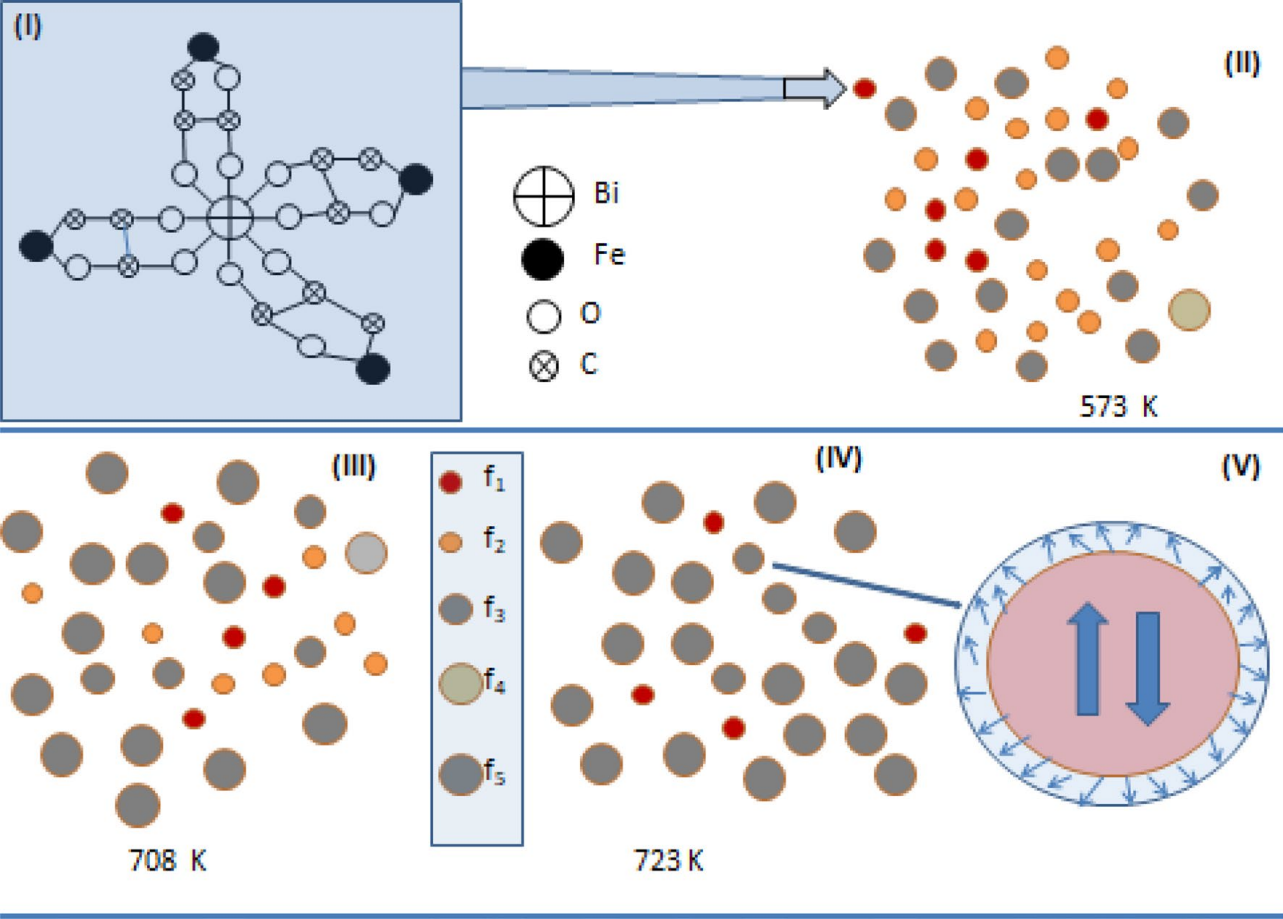
Schematic of the stages involved leading to the nucleation and growth of BiFeO_3_ particles due to calcination of precursor at different temperatures. (I) shows polydentate structure and (II) precursor gel dried at 573 K containing superparamagnetic nanoparticles of BiFeO_3_ and fine nanoparticles of Bi rich/Fe rich oxides as deduced based on Mossbauer results. Each of these particles are formed and stabilized due to bismuth iron tartarate polydentate based polymeric structures terminated by carbon–oxygen bonding network. (iii) Annealing around 700 K leads to a sharp increase in the nanocrystallites of BiFeO_3_ as the fine Bi/Fe rich bismuth iron oxide particles get dissociated and react leading to dominant formation of BiFeO_3_. (iv) Sample annealed beyond 700 K has fine particles of BiFeO_3_ exhibiting AFM core and weakly FM shell as shown in the schematic corresponding to V.

The dried precursor gel as obtained in the sol–gel method is understood to be mainly composed of bismuth iron tartarate as shown in Fig. 7(I). This mainly consists of Bi atoms coordinated with nine oxygen atoms which are in turn connected to Fe atoms using carbon–oxygen chains. As the ionic radius of Bi^3+^ (1.103 Å) is observed to be much higher than that of Fe^3+^ (0.645 Å) there would be a tendency for the formation of these structures with inhomogeneous distribution resulting in zones which are richer in Bi/Fe ions. Importantly these polydentate structures are understood to play an important role in stabilizing the very fine particles of bismuth iron oxide phases which are rich in Bi and Fe respectively due to dangling bonds of C–O chain structures. The C–O chain structures are reported to be stable close to 700 K up to which the emission of CO_2_ has been deduced based on TGA results reported^65,66^. This temperature (700 K) is seen to match with the onset of formation of BiFeO_3_ in large fraction which is reported in the present study in the narrow temperature interval of 700–723 K as discussed earlier. Very fine nanoparticles of BiFeO_3_/alpha-Fe_2_O_3_ with size close to SPM limit as deduced based on the Mössbauer results, are observed to be quite stable as evidenced by the constant value of f_2_ till close to 700 K. The formation of such fine nanoparticles of BiFeO_3_/alpha-Fe_2_O_3_ and their stability are understood to be due to the strain effects due to mismatch in ionic radii of Fe^3+^ and Bi^3+^ and in addition due to defects contributed by C–O dangling bonds which are charged and remain stable close to 700 K.

Annealing of the precursor gel just above 700 K results in the dissociation of these complexes leading to the growth of BiFeO_3_ nanocrystallites. This also enables the reaction between the fine particles of bismuth iron oxides which are richer in Fe and Bi respectively leading to further increase in the fraction of BiFeO_3_ beyond 723 K. Thus the important role played by the bismuth iron tartarate based polydentate structures in terms of strain effects leading to the formation of fine nanoparticles of Bi/Fe rich phases of bismuth iron oxides along with that of BiFeO_3_, thermal stability of these fine nanoparticles due to C–O dangling bonds. Two main processes viz*.,* dominant and rapid growth of nanocrystallites of BiFeO_3_ and reaction between nanoparticles of bismuth iron oxides which are rich Bi/Fe rich phases at temperature close to 700 K leading to the formation of BiFeO_3_. The later process of leads to single phase of BiFeO_3_ as the dissociation temperature of the nanoparticles of bismuth iron oxides rich in Bi/Fe matches with the optimal temperature for the formation of BiFeO_3_. The stability of the fine particles of bismuth iron oxides rich in Bi/Fe is contributed mainly by the bismuth iron tartarate based polydentate structures as discussed. Thus the sol–gel method of preparation of BiFeO_3_ with tartaric acid as the fuel results in the formation of BiFeO_3_ devoid of impurity associated phases though the dried precursor gel was observed to be containing fine nanoparticles belonging to other phases as also evidenced by the results of XRD and Raman spectroscopic studies (Fig. S1 and Table S1). nanoparticles are understood to have got formed in the precursor in the zones with homogeneous distribution of polydentates containing bismuth and iron. Polydentates with the distribution of Fe rich zones are strongly bound and hence are remaining as very fine particles. These are reported to be amorphous^43,44,46,67^.

It is already seen from the results of calcination treatments carried out for 2 h that there is a significantly enhanced dissociation of Bi/Fe rich phases for calcination beyond 700 K leading to the formation of single phased BiFeO_3_ particles. This process is mainly driven by diffusion of Bi/Fe atoms across the surface. It is important to note that for calcinations treatments below 700 K the fractions of Fe atoms associated with doublets (i,e superparamagnetic particles of α-Fe_2_O_3_/BiFeO_3_ and paramagnetic particles of Bi_25_FeO_40_) remain same. As the net doublet fraction remains almost same as that of the precursor it is deduced that diffusion of Fe/Bi across these particles which are rich in Bi/Fe associated phases resulting in the of bismuth iron oxide could not be realized. This is plausibly deduced to be due to appreciable separation of these particles caused by the occurrence of charged defects at the surface of these particles. Electrostatic repulsion of these particles due to charged defects at their surface result in the stability of these fractions of Fe atoms associated with doublets. Importantly the Mössbauer results clearly indicate the possible presence of defects in terms of much lower and higher values of isomer shifts associated with typical components of BiFeO_3_. Similarly the values of isomer shifts corresponding to the fractions f_3_ and f_4_ are also much lower as compared to the typical value of isomer shift corresponding to Fe^3+^^62,68,69^. However there is a decrease in the fraction of Fe atoms experiencing weak ferromagnetic interaction by close to 30%. Concomitantly it is observed that this is equal to the fraction of iron atoms associated with BiFeO_3_. Hence it is understood that the formation of BiFeO_3_ in this case is only due to the coarsening of the weak ferromagnetic BiFeO_3_ nanoparticles. It can be observed that the hyperfine fields and quadrupole splitting as experienced by Fe atoms are much lesser than that of the bulk counterpart.

### Steps involved in the nucleation and growth of Bismuth ferrite

Based on the detailed results of Mössbauer spectroscopic measurements carried out on dried precursor subjected to calcinations at different temperatures, the steps involved in the nucleation and growth of BiFeO_3_ particles are briefly explained with an accompanied schematic illustration (Fig. 7). Bismuth iron tartarate based polydentate structure is shown in Fig. 7(I). Based on the results of Mössbauer studies, the dried precursor gel calcined at 573 K is schematically shown (Fig. 7(II)) to be containing superparamagnetic particles of BiFeO_3_/α-Fe_2_O_3_, fine particles of Bi_25_FeO_40_, nanoparticles of BiFeO_3_ with size ranging above the superparamagnetic limit and well below that of bulk BiFeO_3_ particles. Due to size mismatch in ionic radii of Bi and Fe, there is a tendency for the formation of the nanoparticles of Bi_25_FeO_40_ and alpha-Fe_2_O_3_. Nanoparticles of Bi_25_FeO_40_ are paramagnetically ordered, while the mean size of BiFeO_3_ particles has to less than or close to that of superpara magnetic limit so that the resultant Mössbauer spectrum is doublet.

Figure 7(II) shows the representation based on the Mössbauer results obtained in the precursor subjected to annealing at 573 K in which it is observed that there is a nucleation of BiFeO_3_ phase based on the coalescence of weak ferromagnetic particles. It can be seen from Mössbauer results that the fraction of doublet remains stable for annealing up to 693 K which is mainly understood based on the reasoning that the nanoparticles of Bi_25_FeO_40_ and SPM particles of BiFeO_3_/alpha-Fe_2_O_3_ are terminated with carbon–oxygen bonding of the polydentates thus providing a high stability of these particles. Annealing above 700 K provides the desired activation energy for the reaction between the nanoparticles rich in Bi/Fe leading to the optimal formation of BiFeO_3_ as shown in Fig. 7(III).

Since these nanoparticles which are richer in Bi/Fe display significant surface diffusion of atoms result in effective interaction leading to the formation of nanocrystallites of BiFeO_3_. Weakly ferromagnetic particles of BiFeO_3_/α-Fe_2_O_3_ as nucleating centres for the formation of significantly a large fraction of BiFeO_3_ nanocrystallites as illustrated in Fig. 7(IV). The formation of significant fraction of BiFeO_3_ is enabled by the diffusion of Bi/Fe atoms through the surface of the nanoparticles which are richer in Bi/Fe. The activation energy of formation of BiFeO_3_ particles is observed to be coinciding with the dissociation energy of the fine bismuth iron oxide nanoparticles richer in Bi/Fe with nucleating centres as BiFeO_3_ present in small fraction in stage-I, leading to a significant formation of BiFeO_3_ phase, displayed as stage-II in Fig. 6 showing the variation of fraction of Fe atoms associated with BiFeO_3_ with calcination temperature. Stability of these particles are dictated by the binding of these particles with carbon–oxygen bonds at their surface. Figure 7(V) shows that the nanoparticles of BiFeO_3_ are characterized by the antiferromagnetic core and a weakly ferromagnetic shell. Fractions of Fe atoms (f_5a_, f_5b_) are associated with two distinct Fe sites of FeO_6_ octahedra corresponding to BiFeO_3_ representing antiferromagnetically ordered core while the fraction f_6_ of iron atoms are associated with the shell experiences magnetic interaction as shown in the Table 1. Fe atoms associated with BiFeO_3_ particles of size greater than 70 nm exhibit only AFM ordering. Hence the weak ferromagnetic property is essentially contributed by the shell of fine nanoparticles of BiFeO_3_. Magneto electric coupling effects as observed in the nanoparticles of BiFeO_3_ based on the results of Raman spectroscopy and in terms of P-E loop studies under the application of magnetic field are discussed in the following.

**Figure 8:**
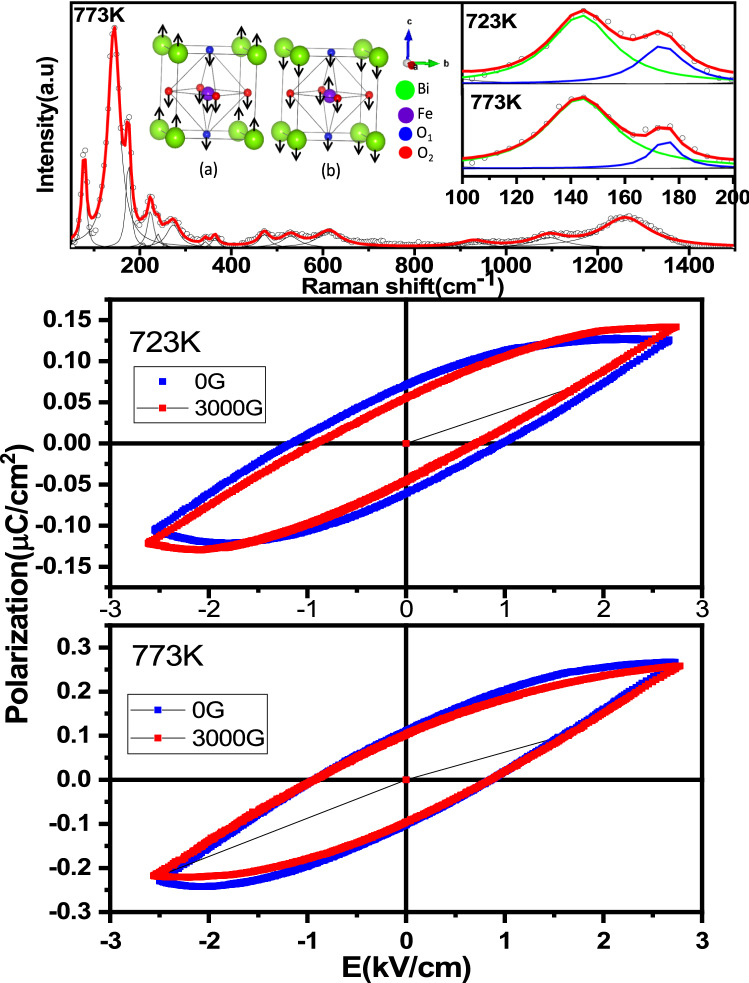
Effect of magneto electric coupling at 300 K in the precursor gel subjected to calcination treatments for 2 h at (**a**) 723 K, (**b**) 773 K.

Top panel of Fig. 8, shows room temperature Raman spectrum obtained in precursor subjected to annealing at 773 K. At room temperature pure BFO belongs to rhombohedral structure with R3c space group with two formulae in one primitive cell having 18 optical phonon modes with irreducible representation

$$ \Gamma_{{{\text{opt}},{\text{ R3c}}}} = {\text{ 4A}}_{{1}} + {\text{ 5A}}_{{2}} + {\text{ 9E}} $$

The A1 (Transverse optical modes) and E (Longitudinal optical modes) are Raman and IR active but A2 modes are Raman inactive. So the irreducible form becomes^70^

$$ \Gamma_{{{\text{Raman}},{\text{ R3c}}}} = {\text{ 4A}}_{{1}} + {\text{ 9E}} $$

Peak fitting of Raman spectroscopy of obtained samples have been done using Lorentzian distributions. We identified 10 modes out of 13 Raman active modes present in the bulk BFO and spectra of each sample is in agreement with those expected for rhombohedral BFO with R3c space group. Raman analysis give indications of spin-phonon coupling in BiFeO_3_^70^. Rhombohedral *R3c* BiFeO_3_ has A1 modes about 136, 168 and 218 cm^−1^. Earlier reports on BiFeO_3_ samples shows phonon mode anomalies during different spin reorientation transition temperatures which give insights on spin-phonon coupling in this system^52,71^. Phonon modes about 136 (A1 1TO) and 168 cm-1 (A1 2TO) are corresponding to Bi-O vibrational modes^72^.

The decrease in peak intensity of this first normal A1 mode (136 cm^−1^) relative to second A1 mode (168 cm^−1^) indicating the suppression in contribution of the Bi-O1 vibrational mode, can most likely be attributed to enhanced coupling of magnetic, ferroelectric, and/or structural order parameters^33,59^. Top panel shows modes corresponding to (a) 136 cm^−1^ and (b) 168 cm^−1^. Raman modes obtained for 773 K and 723 K shows variations in the raltive intensity of A1 1TO (136 cm^−1^) and A1 2TO (168 cm^−1^). The relative intensity I(A1 1TO)/I(A1 2TO) is smaller for 723 K (1.82) than that of 773 K (2.72). Hence, the results of Raman studies indicate that the precursor subjected to calcinations at 723 K exhibits more magneto electric coupling effects than that of the sample calcined at 773 K. It is to be noted that A1 1TO (A2-2TO) mode corresponds to anti phase vibration (in phase vibration) of Bi and O ions. In phase vibrations results in more hybridisation of Bi & O and spin-phonon coupling is more in this mode than out of phase vibration of Bi and O. If the Fe magnetic moments are more coupled with phonon modes, it enhances the amplitude of vibration of in phase vibration of Bi-O ions, which in turn enhances the intensity of A1 2TO mode relative to A1 1TO mode. Hence, the suppression of 136 cm^−1^ mode relative to 168 cm^−1^ indicating enhanced magnetoelectric coupling.

As the precursor is calcined at temperatures above 700 K there is a significant enhancement in the fraction of BiFeO_3_ that gets formed. Coarsening of these particles lead to a situation similar as that of the bulk BiFeO_3_ which has been reported to be exhibiting very less magneto electric coupling effects. Comparison of P-E results as obtained in the precursor subjected to calcination at 723 K with and without the application of an external magnetic field of 0.3 Tesla shows a significant magneto electric coupling effects (Cf Fig. 8). In 723 K annealed sample with the application of magnetic field there is an appreciable decrease in *P*_*r*_ and *E*_*c*_, with an associated increase in *P*_*max*_ as elucidated in the figure. In the precursor annealed at 773 K the observed magneto electric coupling effect is quite small as can be seen.

Atomic scale understanding of the magnetoelectric coupling effect is elucidated based on the comparison of Mössbauer results as obtained in the precursor calcined at 723 K and 773 K respectively . Very fine nanoparticles of BiFeO_3_ are understood to be occuring in core–shell structures with antiferromagnetically ordered BiFeO_3_ core with the outer shell exhibiting weak ferromagnetic nature. Fraction of iron atoms (f_6_) is understood to be associated with shell of nano BiFeO_3_ experiencing weak ferromagnetic interaction with a hyperfine field value close to 42 Tesla. Small fraction of nanoparticles of BiFeO_3_ characterized by the above core–shell configurations are seen to exhibit the observed significant magneto electric coupling effects. The concentration of such nanoparticles in the case of precursor subjected to calcination at 773 K, as characterized by the value of f_6_, is observed to be much lower as compared to that of the precursor annealed at 723 K seen from Mössbauer results.

Importantly the precursor calcined at 723 K with the application of magnetic field shows interesting changes in terms of decrease in quadrupole splitting and isomer shift associated with f_1_. Besides the presence of bulk like BiFeO_3_ particles, there are nanoparticles of BiFeO_3_ present in the system exhibiting core–shell structures having BiFeO_3_ as core and having weak ferromagnetic component at the shell. In addition about 11% of iron atoms ate found to be associated superparamagnetic particles of BiFeO_3_. Also it can be observed that there are appreciable changes in both the quadrupole splitting and hyperfine field with ferromagnetic component (f_6_) subsequent to calcination at 723 K. It is observed that the values of hyperfine parameters remain largely the same in the case of calcined sample at 773 K in the cases of with and without the application of magnetic field viz*.*, no appreciable change in the values of hyperfine parameters associated with f_1_ and the weak ferromagnetic component (f_6_). Thus Mössbauer results clearly elucidates the importance of nano nature of BiFeO_3_ and associated defect structures for the system to exhibit weak ferromagnetic and the strong magneto electric coupling effects.

It is already seen from P-E results that the ferroelectric effect is significantly higher in the case of precursor annealed at 723 K than that of 773 K. Fraction associated with weak ferromagnetic interaction exhibits significant changes in terms of hyperfine field value with the application of magnetic field. The weak ferromagnetic component as contributed by the shell of the nanoparticles of BiFeO_3_ is understood to be due to un compensated surface spins, strain effect leading to significantly reduced value of Fe–O–Fe angle as compared to that of the bulk BiFeO_3_^35,73–78^. Increase in the magnetic hyperfine field associated with the weak ferromagnetic component of the shell of BiFeO_3_ with the application of external magnetic field is much higher in the case of the precursor subjected to calcination at 723 K as compared to that of 773 K calcined sample.

**Figure 9:**
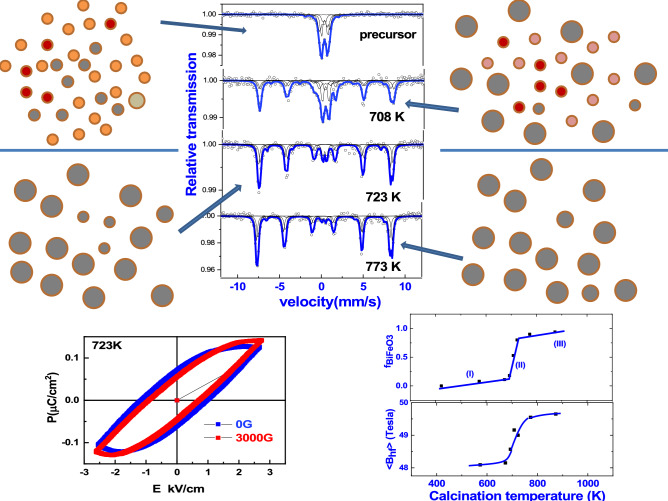
*Pictorial summary of the results of this work* showing the effect of calcination of the precursor gel obtained using sol–gel method of preparation of BiFeO_3_ with tartaric acid as the fuel based on Mӧssbauer studies. Mӧssbauer results in the dried precursor gel reveal the presence of SPM particles of BiFeO_3_ along with the very fine paramagnetic nanoparticles of Bi_25_FeO_40_. Mӧssbauer results imply the coalescence of nanoparticles of BiFeO_3_ marked as stage-I, followed by significant growth of nanocrystallites of BiFeO_3_ in stage-II over a narrow temperature interval of 700–723 K, with stage-III is understood to be due to coarsening of nanocrystallites in to polycrystallites of BiFeO_3_. Significant magneto electric coupling effect is elucidated in the precursor gel calcined at 723 K. Increase in the value of the mean hyperfine field at Fe sites in BiFeO_3_ due to increase in the size of nanocrystallites of BiFeO_3_ as caused by calcination is also shown.

Summarizing this study elucidates the nucleation and growth of nanocrystallites of phase pure BiFeO_3_, prepared through sol–gel route with tartaric acid as fuel, at atomic scale based on the studies on the evolution of local structural and magnetic properties at Fe sites. Polydentate structure having metallic ions such as Bi, Fe get formed in the sol–gel process. Because of larger size of Bi ion as compared to Fe ions there is a tendency for the inhomogeneous distribution of Bi, Fe ions in polydentates leading to the formation of Bi rich/deficient zones. The super paramagnetic particles of BiFeO_3_/α-Fe_2_O_3_ and fine particles of Bi_25_FeO_40_ remain quite stable up to 700 K implying that these particles are strongly stabilized by polydentates with C–O bonding. Beyond 700 K following the dissociation of C–O bonding these particles react quite strongly leading to the growth of BiFeO_3_ with the single crystallite nanoparticles of BiFeO_3_ acting as nucleating centres for the growth. Interestingly the temperature of dissociation of these polydentatesis understood to match quite closely with the temperature of reaction of these phases leading to the formation of maximum concentration of BiFeO_3_. Results of the Mössbauer study thus nicely elucidate the local structural, magnetic properties of Fe atoms associated with the particles of different phases thus providing an atomic scale understanding of the evolution of the nucleation and growth of BiFeO_3_. Important results of this study as summarized are represented graphically in Fig. 9. Further the present Mössbauer study brings out the important contribution of defects associated with the nanoparticles of BiFeO_3_ for the observed weak ferromagnetic and hence magneto electric coupling effects as observed.

## Conclusions

This study provides an atomic scale understanding of the formation of phase pure BiFeO_3_ by means of studying the evolution of the local structural and magnetic properties at Fe sites subsequent to detailed calcination treatments of the precursor derived from sol–gel method with tartaric acid as the fuel using Mossbauer spectroscopy. BiFeO_3_ nanocrystallites thus formed are observed to exhibit weak ferromagnetic and ferroelectric ordering. Results of the Mossbauer study shows the presence of superparamagnetic particles of BiFeO_3_/α-Fe_2_O_3_, nanoparticles of BiFeO_3_ experiencing weak ferromagnetic interaction along with the nanoparticles of Bi_25_FeO_40_ in the precursor gel. Three important stages have been deduced with the dominant growth of nanocrystallites of BiFeO_3_ occurring in the calcination temperature interval of 700–723 K. Annealing beyond 730 K results in the coarsening of these crystallites leading to the formation of polycrystalline BiFeO_3_. Mossbauer results obtained in the sample calcined at 723 K under the application of magnetic field show appreciable changes in the hyperfine parameters corresponding to weak ferromagnetically ordered shell of the nanoparticles of BiFeO_3_ as compared to the results obtained in the absence of external magnetic field. These results bring out the importance of the defect structure associated with the weak ferromagnetically ordered shell of BiFeO_3_ playing a significant role for the observed magneto electric coupling effects. The results of this study might be quite useful in envisaging various applications of BiFeO_3_ by means of fine tuning the defects and hence the magneto-electric coupling effects.

## Supplementary Information

Supplementary Information.
