# Supplementary material for: Atomic scale insights on the growth of BiFeO3 nanoparticles
Source: Sci Rep. 2022 Mar 19;12:4758. doi: 10.1038/s41598-022-08687-y (PMC8934348; doi:10.1038/s41598-022-08687-y)
Supplement: Supplementary file 1 — Supplementary Information. [file 41598_2022_8687_MOESM1_ESM.docx]

Supplementary Information

**Atomic scale insights on the growth of BiFeO_3_ nanoparticles**

Parvathy N. S and R. Govindaraj*

Materials Science Group

Indira Gandhi Centre for Atomic Research, HBNI, Kalpakkam – 603102 , Tamil Nadu, INDIA

**Figure S1.** XRD patterns and Raman spectra as obtained in the precursor gel calcined at different temperatures are shown in left and right panels respectively. These patterns are de-convoluted to identify different phases present


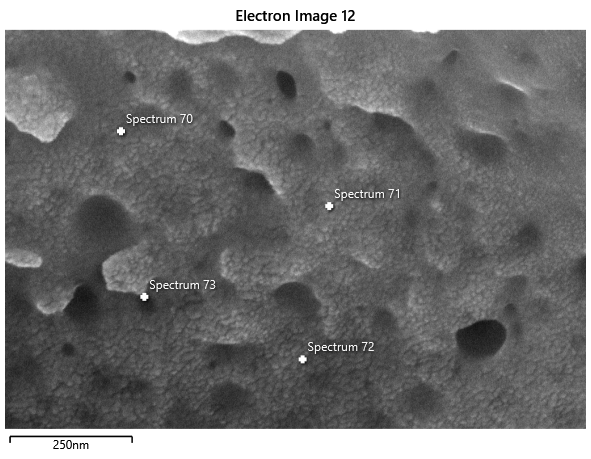





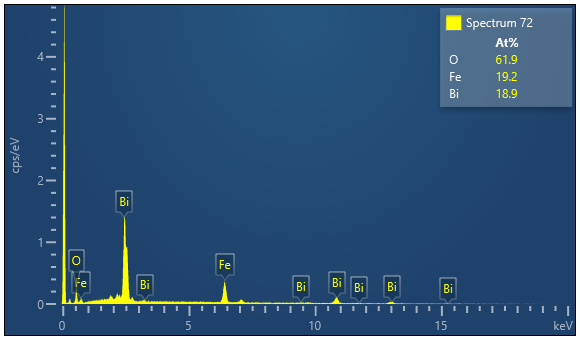


**Figure S2.** Top panel shows Scanning Electron Microscopy (SEM) micrographs obtained in the precursor subjected to calcination treatment at 573 K for 2 hours. SEM micrograph shows the presence of BiFeO3 particles of mean size close to 15 nm . Bottom panel shows a representative EDAX pattern obtained at a spot in the sample clearly showing the presence of bismuth iron oxide nanoparticles present in almost stochiometric ratio close to 1:1:3.

**Table S1**. Identification of different phases present in the precursor gel subsequent to calcination for 2 hours at different temperatures viz., 673 K and 773 K as deduced based on the de-convolution of the respective XRD patterns and Raman spectra ^[1-7]^  as shown in Figure S1.

| Calcination temperature of precursor gel | XRD peak positions (degrees) | Raman modes (cm^-1^) | Phase identification |
| --- | --- | --- | --- |
| 673K | 27.7 , 30.2 | 157, 434 | Bi_25_FeO_40_ |
|  | 31.78 , 31.85, 32.62 | 76, 137, 168, 603, 1261 | BiFeO_3_ |
|  | 33.17 | 300, 490, 555, 649, 703 | Fe_2_O_3_ |
| 773K | 31.74, 31.97 | 78, 143, 174, 223, 273, 343, 364, 472, 529, 615, 942, 1088, 1258 | BiFeO_3_ |

**Table S2. Compilation of the results of the some of the reported results (not exhaustive) on the synthesis of nano BiFeO_3_ through chemical route**

| Experimental details | Fuel and complexing agent (if any) | Research highlights | Literature |
| --- | --- | --- | --- |
| - Solvent evaporation method   Bi(NO_3_)_3_.5H_2_O  Fe(NO_3_)_3_.9H_2_O  HNO_3_ | Tartaric acid | - Phase pure BiFeO_3_ has been formed at 723K - Asymmetric amorphous XRD peak obtained in samples calcined below 723K | [8] |
|  | Tartonic acid | - 773K sample XRD shows BiFeO_3_ as majority phase along with Bi_25_FeO_39_ - Samples calcined below 723K doesn’t have crystalline BiFeO_3_ phase |  |
|  | Mucic acid  Combustion at 433K | - Crystallization of BiFeO_3_ happened T_cal_<623K - Presence of Bi_25_FeO_39_ in samples calcined upto 773K with amorphous surface layer as TEM result - High M_s_ value at 1T due to magnetic impurities |  |
|  | Mucic acid and Nacl | - NaCl salt decreases crystallization temperature, enhances phase purity and monocrystalline nature - Ms value at 1T decreased with NaCl addition |  |
| - Pechini method   Bi(NO_3_)_3_.5H_2_O  Fe(NO_3_)_3_.9H_2_O  HNO_3_ | Maleic acid + Ethylene glycol | - Phase pure BiFeO_3_ obtained in samples calcined between 683 K to 873K,sizes (d) varies from 13nm to 72nm - Below 30nm, lattice deviate from bulk and approaching cubic perovskite structure for d<30nm - T_N_ decreases significantly below bulk value for d<50nm | [9] |
|  | Malic acid | - 773K and 873K calcined sample shows phase pure BiFeO3 with size varies from 20nm to 86nm - T_cal_<773K shows amorphous nature and T_cal_>873K shows Bi_2_Fe_4_O_9_ phase |  |
|  | Malic acid + Ethylene glycol | - 873K sample shows phase pure BiFeO_3_ |  |
|  | Tartaric acid | - 873K sample shows Bi_2_Fe_4_O_9_ phase as impurity |  |
| Sol gel  Bi(NO_3_)_3_.5H_2_O  Fe(NO_3_)_3_.9H_2_O  HNO_3_ | Ethylene glycol | - 773 K sample XRD shows phase pure BiFeO_3_ with d=31nm - Samples obtained at T_cal_> 773K has impurity phases - 0.05 emu/g M_s_ at 4kOe with negligible loop area | [10] |
| Sol gel  Bi(NO_3_)_3_.5H_2_O  Fe(NO_3_)_3_.9H_2_O  HNO_3_ | Tartaric acid | - Phase pure BiFeO_3_ obtained in samples calcined at 723K <T_cal_< 798 K with size varies from 18nm to 83nm - Weak ferromagnetic property - Magnetization increases with size reduction and showing peculiar magnetism for 62nm particle. | [11] |
| Sol gel  Bi(NO_3_)_3_.5H_2_O  Fe(NO_3_)_3_.9H_2_O  HNO_3_ | Tartaric acid | - 873K calcinations resulted phase pure BiFeO3 phase - Precursor powder from synthesis displayed amorphous XRD pattern before calcination | [12] |
|  | Tartaric acid + Ethylene glycol | - Weak unidentified reflections obtained for powders before calcinations - 873K calcined samples shows Bi_2_Fe_4_O_9_ as impurity |  |
|  | Malic acid | - Observed weak unidentified peaks for powders before calcinations and it became amorphous when T_cal_= 673K - 773K and 873K calcined samples shows BiFeO_3_ phase |  |
|  | Malic acid +Ethylene glycol | - Precursor was amorphous and 873K sample XRD shows phase pure BiFeO3 |  |
|  | Succinic acid | - 873K calcination shows BiFeO_3_ phase with impurity |  |
|  | Succinic acid + Ethylene glycol | - 873K calcination shows BiFeO_3_ phase with impurity |  |
|  | Maleic acid | - Weak unidentified reflections obtained for powders before calcinations - 873K calcined samples shows impurities |  |
|  | Malonic acid | - Unsuccessful in synthesising phase pure BiFeO3 at 873K calcination |  |
|  | Malonic acid + Ethylene glycol | - Weak unidentified reflections obtained for powders before calcinations - Unsuccessful in synthesising phase pure BiFeO3 at 873K calcination |  |
| Pechini’s auto combustion method  Bi(NO_3_)_3_.5H_2_O  Fe(NO_3_)_3_.9H_2_O  HNO_3_ | Citric acid + Ethylene glycol | - 873K calcined sample shows β- Bi_2_O_3_, BiO, Bi_25_FeO_40_ and Bi_46_Fe_2_O_72_ as impurity phases | [13] |
| Low temperature chemical route Bi(NO_3_)_3_.5H_2_O  Fe(NO_3_)_3_.9H_2_O  HNO_3_ | Oxalic acid | - 693K calcined sample shows BiFeO_3_ as majority phase with small fraction of impurity and size d=100nm - Weak ferromagnetic property with RT M_s_ value of 0.27emu/g at 20kOe with finite coercivity of 156Oe | [14] |
|  | Oxalic acid + Oleic acid | - Oleic acid stabilization decreased particle size to 40nm - 693K calcined sample has BiFeO_3_ as majority phase with small fraction of impurity - RT M_s_ value enhanced to 4.39emu/g with H_c_ = 137Oe |  |
|  | Tartaric acid + Ethylene glycol | - 773K sample shows phase pure BiFeO_3_ phase - T_cal_=873K and 1073K promotes Bi_25_FeO_40_ formation - T_cal_= 673K and 723K shows Bi_2_O_3_ as impurity - Weak ferromagnetic property with magnetization enhancement as particle size decreases. | [15] |
| Sol gel  Bi(NO_3_)_3_.5H_2_O  Fe(NO_3_)_3_.9H_2_O  HNO_3_ | Tartaric acid | - T_cal_=723 K and 773 K sample shows the formation of pure BiFeO_3_ as deduced by XRD - Annealing at 873 K and beyond promotes Bi_25_FeO_40_ formation - Weak ferromagnetic property with magnetization is observed to increase with decreasing particle size | Present work |

**References**

1 Hermet, P., Goffinet, M., Kreisel, J. & Ghosez, Ph. Raman and infrared spectra of multiferroic bismuth ferrite from first principles. *Phys. Rev. B* **75**, 220102 (2007)

2 Fukumura, H. *et al.* Observation of phonons in multiferroic BiFeO_3_ single crystals by Raman scattering. *J. Phys.: Condens. Matter* **19**, 365224 (2007)

3 Muneeswaran, M & Giridharan, N. V. Effect of Dy- substitution on the structural, vibrational, and multiferroic properties of BiFeO_3_ nanoparticles. *J. Appl. Phys.* **115**, 214109 (2014)

4 Zhang, L. *et al.* Hydrothermal synthesis, influencing factors and excellent photocatalytic performance of novel nanoparticle – assembled Bi_25_FeO_40_ tetrahedrons. *Cryst Eng Comm.* **17**, 6527- 6537 (2015).

5 Wu, Y., Luo, H., Jiang, X., Wang, H. & Geng, J. Facile synthesis of magnetic Bi_25_FeO_40_/rGO catalyst with efficient photocatalytic performance for phenolic compounds under visible light. *RSC Adv.* **5**, 4905- 4908 (2015)

6 López-Sánchez, J. *et al.* Sol-gel synthesis and micro-Raman characterization of ε-Fe_2_O_3_ micro and nanoparticles. Chem. Mater. **28**, 511- 518 (2016)

7 López-Sánchez, J. *et al.* Growth, structure and magnetism of ε-Fe_2_O_3_ in nanoparticle form. *RSC Adv.* **6**, 46380-46387 (2016)

8 Clarke, G. *et al.* Preparation from revisited wet chemical route of phase-pure monocrystalline and SHG- efficient BiFeO_3_ nanoparticles for harmonic imaging. *Sci. Rep.* **8**, 10473 (2018).

9 Selbach, S. M., Tybell, T., Einarsrud, M. A. & Grande, T. Size – dependent properties of multiferroic BiFeO3 nanoparticles. *Chem. Mater.* **19**, 26, 6478-6484 (2007)

10 Diliautas, R. et al. Reinspection of formation of BiFe_1-x_Mn_x_O_3_ solid solutions via low temperature sol gel synthesis route. *Solid state sci.* **111**, 106458 (2021)

11 Huang, F. *et al*. Peculiar magnetism of BiFeO_3_ nanoparticles with size approaching the period oft the spiral spin structure. *Sci. Rep*. **3**, 2907 (2013)

12 Selbach, S. M., Einarsrud, M. A., Tybell, T. & Grande, T. Synthesis of BiFeO_3_ by wet chemical methods. *J. Am. Ceram. Soc.* **90**, 3430 - 3434 (2007)

13 Ghosh, S., Dasgupta, S., Sen, A. & Maity, H. S. Low temperature synthesis of nanosized bismuth ferrite by soft chemical route. *J. Am. Ceram. Soc.* **88**, 5, 1349 – 1352 (2005)

14 Mahesh, D., Mandal, S. K., Mahato, B. K., Rana, B. & Barman, A. Pronounced multiferroicity in oleic acid stabilized BiFeO_3_ nanocrystals at room temperature. *J Nanosci. Nanotechnol.* **13**, 4090-4096 (2013)

15 Pikula, T. *et al.* The influence of annealing temperature on the stucture and magnetic properties of nanocrystalline BiFeO_3_ prepared by sol gel method. *Metall Mater Tran A* **53**, 470-483 (2022)
